# Eliminating episodic memory?

**DOI:** 10.1098/rstb.2023.0413

**Published:** 2024-09-16

**Authors:** Nikola Andonovski, John Sutton, Christopher Jude McCarroll

**Affiliations:** ^1^ Centre for Philosophy of Memory, IPhiG, Université Grenoble Alpes, Saint-Martin-d’Heres 38400, France; ^2^ Philosophy, Macquarie University, Sydney, New South Wales, Australia; ^3^ Philosophy, University of Stirling, Stirling, UK; ^4^ Institute of Philosophy of Mind and Cognition, National Yang Ming Chiao Tung University, Taipei, Taiwan

**Keywords:** episodic memory, memory systems, eliminativism

## Abstract

In Tulving’s initial characterization, episodic memory was one of multiple memory systems. It was postulated, in pursuit of explanatory depth, as displaying proprietary operations, representations and substrates such as to explain a range of cognitive, behavioural and experiential phenomena. Yet the subsequent development of this research programme has, paradoxically, introduced surprising doubts about the nature, and indeed existence, of episodic memory. On dominant versions of the ‘common system’ view, on which a single simulation system underlies both remembering and imagining, there are no processes unique to memory to support robust generalizations with inductive potential. Eliminativism about episodic memory seems to follow from the claim that it has no dedicated neurocognitive system of its own. After identifying this under-noticed threat, we push back against modern eliminativists by surveying recent evidence that still indicates specialized mechanisms, computations and representations that are distinctly mnemic in character. We argue that contemporary realists about episodic memory can retain lessons of the common system approach while resisting the further move to eliminativism.

This article is part of the theme issue ‘Elements of episodic memory: lessons from 40 years of research’.

## 1. Introduction

‘Most, if not all, of our currently held ideas and theories about mental processes are wrong’, Tulving admitted in his 1984 APA award address, and ‘sooner or later in the future they will be replaced with more adequate concepts, concepts that fit nature better’ [1, p. 386]. So, it was with modesty as well as ambition that, in *Elements of episodic memory* [2], he sought to bring that future closer, characterizing episodic memory as a specialized neurocognitive system underlying the capacity for remembering the personal past. It is the possession of this system that allows humans—indeed, only humans—to mentally travel back into the past and re-experience it in that warm and intimate way William James had described a century before.

Four decades after *Elements,* the sheer ubiquity of the concept of episodic memory may seem a reliable indicator of its good fit with nature. Textbooks and review articles routinely characterize episodic memory as a core mental capacity. The most notable development in the field, itself spearheaded by Tulving’s pioneering work, has been the discovery of a close processing connection between remembering and imagining, often taken to indicate that a single neurocognitive system underlies both. Yet the deceptively simple shift to this ‘common system’ view introduces surprising doubts about the nature, and indeed existence, of episodic memory. We ask whether the new theories imply that the concept of episodic memory does not, after all, ‘fit nature’ well. Is the natural scientific development of Tulving’s concept paradoxically leading to its elimination?

After summarizing the main features of Tulving’s [2] original theory of episodic memory, we argue that on some interpretations this new generation of theories, positing a common system for remembering and imagination, lead to eliminativism: on such theories, episodic memory may not be a real kind. The consequences of this under-noticed implication may be severe. Eliminating episodic memory would potentially have dramatic costs, both practical and scientific, disrupting both our normative reliance on memory in social, legal and emotional contexts, and our empirical progress towards theories aiming at unification and consilience [3–5]. Given that the stakes are high, we should proceed with caution when faced with eliminativism. Retaining episodic memory may facilitate the integration of research on engrams, systems, learning, phenomenology and perhaps even autonoetic consciousness.

Having identified the threat, we go on to synthesize recent evidence, from various research programmes in the memory sciences, that challenges the eliminativist conclusion. This evidence, we argue, should make us reasonably optimistic about the existence of specialized episodic memory processes, potentially vindicating the core idea of *Elements*. We conclude by sketching options for revised forms of realism about episodic memory that still take on board the recent developments.

## 2. Tulving’s *Elements*: episodic memory as an explanans

Tulving developed his theory of episodic memory, first systematically presented in *Elements,* within the multiple memory systems approach, which had emerged as a dominant research framework by the end of the 1970s.^1^ On this approach, memory is not a unitary mental faculty but is composed of a number of memory systems: functionally distinct neurocognitive structures, constituted by sets of processes with proprietary principles of operation, representational kinds and neural substrates [7,8]. Memory systems constitute the basic *kinds* of memory and underpin the abilities of organisms to acquire, retain, retrieve and use information in a variety of productive ways. Episodic memory, Tulving argued, is a dedicated memory system that underpins the ability of individuals to remember events from their personal past and to re-experience them in a quasi-perceptual way, ‘travel[ing] back into the past in their own minds’ [2, p. 1]. Episodic memory is functionally distinct from semantic memory—a system underlying an individual’s general or impersonal knowledge of the world—as well as from a number of procedural memory systems supporting the acquisition of various motor, perceptual and cognitive skills.

From the perspective of philosophy of science [9], the pursuit of explanatory depth lies at the heart of the multiple systems approach. The systems theorist aims to look beyond ‘surface’ variety to offer explanatory accounts of the underlying neurocognitive structures whose workings produce introspectively and behaviourally identifiable memory phenomena. In doing so, they aim to articulate principles that unify a variety of empirical generalizations, often from very different experimental paradigms. Such principles, along with the computational and representational properties of memory systems, account for the phenomena characterized by the available behavioural and phenomenological data. Memory systems, to employ a familiar philosophical idiom, are *natural* kinds [10,11]. They are causally organized clusters of properties of encoding, consolidation, storage and retrieval.^2^ By design, the instantiation of some of these properties tends to cause the instantiation of the others, resulting in a robust and systematic correlation between them. By virtue of being natural kinds, memory systems afford productive theorizing, with natural kind terms like ‘episodic memory’ featuring in a wide range of stable empirical generalizations. They are thus to be distinguished from merely ‘descriptive forms of memory’—such as verbal memory or recognition memory—which help describe and organize empirical facts but which do not carry such inductive potential [7, pp. 11–12].

Hypothesized to operate in a proprietary way, memory systems can be theorized about independently—in idealized isolation from their interaction with other systems. Yet, in reality, systems like episodic and semantic memory ‘are closely interdependent and interact with one another virtually all the time’ [2 p. 65]. Such interactions, which can be cooperative or competitive, occur in the production of most individual memories and are manifested in the performance of even simple experimental tasks. The relation between memory systems and memory tasks is many-to-many [2,7,12].

In *Elements*, episodic memory was thus posited as an explanans (that which does the explaining), its distinctive properties intended to account for core aspects of relevant memory phenomena. The theory sought to unify explanations of two main classes of such phenomena. The first is the ability of subjects to retain information about the properties of, and spatio-temporal relations between, items acquired on singular learning occasions, as manifested in (for example) classic verbal learning experiments [13]. The second, more prominently, concerned the phenomenology of remembering, which Tulving, following tradition, took to be marked by a ‘definite affective tone that is uniquely and unmistakably one of the salient attributes of recollective experiences’ [2, p. 48]. When remembering events from the personal past, subjects are immediately aware that they are re-experiencing events they have experienced before, a kind of awareness Tulving [14] would label autonoetic (i.e. self-knowing).^3^ As a result, they are disposed to assert epistemic authority with respect to them, claiming knowledge of what happened even when unsure about the events’ precise spatio-temporal location. This ‘subjective veridicality’, characteristic of personal recollection, is largely absent when we recall impersonal facts [2, p. 40].

We highlight three properties of the episodic memory system that Tulving introduced to account for these phenomena. First, the system was hypothesized to possess a proprietary store, in which information—paradigmatically, about the perceptible properties of experienced events—is registered directly [2, pp. 41–42]. Second, unlike semantic memory, episodic memory was taken to be relatively limited in its capacity to generate novel information not already present in the input [2, pp. 43–44]. Third, episodic retrieval was seen as a synergistic process, necessarily involving ‘mixing’ of information in the store, i.e. trace information, and information in the retrieval environment [2, pp. 171–176].^4^ When the system functions properly, the retention of information encoded in the store affords a kind of ‘immediate, or first-hand knowledge’ of previously experienced events [2, p. 41], allowing subjects to successfully perform a variety of tasks that require such retention. Moreover, since the system is limited in its capacity to generate novel information, the very presence of some information in the store (at the point of retrieval) indicates that it was acquired on an epistemically relevant prior occasion. This partially grounds subjects’ autonoetic awareness of a remembered event. Indeed, Tulving [14] argued, such awareness varies with the nature of the ‘mix’ between trace and cue information at retrieval: the larger the proportion of trace information, the greater the degree of autonoetic awareness.

In the pursuit of explanatory depth, *Elements* posited a specialized episodic memory system, whose functioning involved a delicate interaction between processes of information storage and dynamic, context-sensitive retrieval. The theory characterized episodic memory as a neurocognitive kind, supporting productive theorizing and causal generalizations with inductive potential. The theory, however, was not a taxonomic proposal for the classification of individual memor*ies*. Most memories emerge via the systematic interaction of multiple memory systems, a fact that led Tulving to characterize the task of unambiguously classifying a particular memory as episodic or semantic as ‘uninteresting and lead[ing] nowhere’ [15, p. 5]. The systems theorist aims to account for essential elements of memory phenomena—some of which, like the nature of conscious retrieval, may reflect underlying systemic differences—yet is fully aware of their behavioural and phenomenal diversity.^5^

## 3. Tulvingian eliminativism: from episodic memory to mental time travel but *not* back

Forty years after *Elements,* episodic memory has remained a key notion in the sciences of memory. Tulvingian themes dominate the contemporary discussion, as evidenced by the steady and well-documented rise in the number of articles dedicated to the examination of episodic memory, autonoetic consciousness and mental time travel [16–18]. A number of empirical developments have nevertheless gradually driven a notable and significant shift in focus.

Unsurprisingly, Tulving played a key role in initiating this new research. In 1985 [14], he reported the case of K.C., who had suffered a severe head injury in a traffic accident, resulting in widespread brain damage that included large bilateral hippocampal lesions. As a result, K.C. displayed a profound ‘episodic’ amnesia, being unable to recollect any personally experienced events from his past.^6^ Intriguingly, he was also incapable of *imagining* possible future events, which suggested a close processing link between episodic memory and imagination [14]. The link was confirmed by subsequent neuropsychological studies reporting deficits exhibited by hippocampal amnesiacs in the imagination of novel events, whether located in the future or in the possible past [20–22]. In a parallel development, a slew of neuroimaging studies revealed that both remembering and imagination engage the default mode network (DMN), comprising the medial and lateral temporal lobes (hippocampus included), the medial prefrontal cortex, the posterior cingulate cortex and the inferior parietal lobule [23–25].^7^ Psychological research also revealed developmental parallels between remembering and imagining [27] as well as analogous effects of temporal proximity and mental imagery abilities [28,29].

A new generation of theories, positing a common system underlying remembering and imagination—and, potentially, a number of other capacities—have emerged as a result. Tulving [30] again led the charge himself, characterizing episodic memory as a neurocognitive system for ‘mental time travel’ through subjective time. The operations of the system, he argued, allow subjects to re-experience the personal past in memory and pre*-*experience the personal future in imagination; both instances of autonoetically flavoured mental time travel (cf. [31]). Rival theories share the commitment to a common system, yet provide alternative accounts of its core operations. Schacter & Addis [32] characterize the system as one for ‘constructive simulation’ of possible events—in the past *and* future—a process that typically involves recombination of informational details acquired in the subject’s previous experience (see also [26,33]). In the same spirit, Hassabis & Maguire [34] see remembering and imagination as underlaid by a system for the generation and maintenance of spatially coherent ‘scene’ representations (compare also [35,36]).

Importantly, the new theories inherit the spirit and methodological commitments of the multiple memory systems approach. Indeed, the positing of a common system—for mental time travel, constructive simulation or scene construction—is driven by the pursuit of core computational principles that may underlie a variety of cognitive activities and unify different empirical generalizations. The system, hypothesized to function in accordance with such principles, produces the behaviourally and introspectively accessible properties of personal remembering as well as future-oriented and counterfactual imagination. Proponents of these theories are happy to, and indeed often do, talk of episodic memory. Up to this point, we see continuities between the earlier and newer approaches.

But in fact, a significant terminological shift has gradually emerged, albeit often unnoticed, opening the door to doubts about the very existence of the system Tulving had originally sought to characterize. ‘Episodic memory’ is now most frequently used to refer to a product-state of the relevant system and *not* to the system itself. To put the point somewhat provocatively, ‘episodic memory’ now typically refers to the explanandum (that which needs to be explained), not the explanans. Addis [26, p. 70] provides a representative example, when she suggests that ‘instead of conceptualising imagination as relying on episodic memory, we should consider memory and imagination as but two products of a constructive simulation system’ (see also [34,37,38], among many others). We might decide to brush this off. After all, as Tulving repeatedly emphasized throughout his career, ‘memory’ can be used to refer both to a neurocognitive system and to its typical product. The shift, however, obscures a more significant issue: the dramatic change in the nature of the explanans.

On the picture presented in *Elements,* the operations of the episodic memory system were of direct explanatory significance for core properties of the target mnemic phenomena. This directness, indeed, licensed a verdict on function: an information-preserving system with limited generative capability seemed tailor-made for providing first-hand knowledge of personally experienced events. As theories shifted to a common system underlying a variety of more disparate phenomena, the hypothesized operations became more inferentially distant from (descriptions of) these phenomena (see [9, §4]). While details vary, four important changes are worth highlighting. First, the new theories do not typically posit a proprietary ‘episodic’ store, dedicated to the retention of information acquired on singular, epistemically relevant occasions. Rather, the system is considered to utilize information from a variety of sources in a flexible and context-sensitive manner. Second, the system is seen as strongly generative, routinely and systematically producing information not present in the original input. Third, and relatedly, episodic retrieval is characterized as a more thoroughly reconstructive process, involving the formation of distinct kinds of representations, carefully calibrated for a variety of mnemic and imaginative uses. The process, typically seen as simulational in nature, is taken to constitutively depend on the operations of the DMN. Given that the DMN seems to be involved in a range of episodic simulations, this structure is understood as ‘the brain’s simulation system’ [26, p. 71], and episodic remembering is taken to be one particular operation of this system.^8^ Finally, there has been a significant shift in the characterization of the system’s proper function, now systematically linked to the enhancement of subjects’ predictive prowess and ability to imagine the future. The episodic system, in short, is not really *for* remembering the personal past (for discussions, see [26,30–35,38–41]).

Crucially, the mental time travel/simulation system is hypothesized to function uniformly, with the same set of neurocognitive operations underlying personal remembering and different forms of imagination. This *identity-of-process* hypothesis, widely accepted by systems theorists, is nicely articulated by Addis ([26, p. 70], emphases added):^9^

From a neurocognitive perspective, it is likely that an event representation is physically instantiated in the brain in *exactly the same way irrespective of whether it is remembered or imagined:* it is a set of connections between the nodes representing perceptual content, semantic information and related schemas... [In remembering and imagining events] *the same simulation process is engaged and the same types of information are drawn upon*.

Hence, not only does a common neurocognitive system underlie both remembering and imagining; further, there are no component processes of the system dedicated solely to the production of memories or imaginings. Both involve the same strongly generative process of reconstructive retrieval in which information from different sources is pieced together in an event representation that aims to satisfy a number of constraints (such as relevance, accuracy, general plausibility, consistency with knowledge of past/future events). The identity-of-process hypothesis does not preclude the existence of *some* differences between memories and imaginings, likely resulting from the task-sensitive differential engagement of the constituent operations of the system. These, however, are differences in degree and not in kind [25,26,33,38,39].

These developments turn the new systems theories decisively in the direction of eliminativism about episodic memory. Tulving’s original ambition of accounting for autonoetically flavoured remembering by appeal to a specialized *memory* system seems to have been given up [9]. At least prima facie, a neurocognitive system for mental time travel or constructive simulation—lacking a dedicated store and selected for its effects on subjects’ ability to imagine the future—is not such a system. Moreover, given identity-of-process, there is no specialized component process of this system uniquely engaged during personal remembering. The consequences are far-reaching. The multiple systems approach had treated memory systems and their constituent processes as constituting the basic memory kinds, which afford productive and inductively fruitful theorizing. But if, in contrast, the identity-of-process hypothesis held by common system theorists is correct, then episodic memory is *not* a real kind; indeed, it is not even a sub-kind of mental time travel or constructive episodic simulation. As a result, there are no causal generalizations about episodic memory that carry inductive potential.

This striking conclusion has indeed surfaced in recent philosophical discussions: in particular, Michaelian’s [38] variant of the simulation theory has been diagnosed as eliminativist [44,45]. Such eliminativism about episodic memory does not entail that humans do not engage in remembering the personal past, a mental activity that may exhibit a number of unique characteristics. It does, however, entail that there is no dedicated neurocognitive episodic memory system that underlies remembering. In this regard, remembering may be less like visual perception and more like cherishing, overlooking or disregarding.^10^

We should be careful here. An eliminativist’s modus ponens is a revisionist’s modus tollens. A revisionist, looking at the new developments, may insist on identifying episodic memory with the ‘broader’ neurocognitive system. Indeed, this is the route taken by Tulving [30, p. 9] who argued that episodic memory just *is* a system that ‘makes possible mental time travel through subjective time—past, present, and future’.^11^ This route is certainly open. As generations of philosophers have learned from Quine, an empirical discovery can be redescribed as a change in meaning. Our goal here is thus not to legislate the use of ‘episodic memory’ in the sciences of memory. It is rather to highlight the significant changes in theorizing about the system hypothesized to support personal remembering. These involve a shift from an information-preserving system that affords first-hand knowledge of the personal past to a more general system for event representation whose evolutionary function may indeed not be mnemic at all. We take the characteristic reluctance of theorists to use ‘episodic memory’ to refer to such a system as a reliable indicator that this shift is indeed quite significant. A revisionist who takes this lesson on board is, for our purposes at least, not essentially different from an eliminativist about episodic memory. To be clear, we do *not* think that the common system hypothesis entails eliminativism. In fact, we take it as plausible that some forms of common system theory will retain some form of realism about episodic memory, thus resisting eliminativism: there are a range of less radically revisionary ways to incorporate the key lessons of common system theories. But our argument here is, nevertheless, that the currently predominant versions of common system theory do in fact eliminate episodic memory as a real kind.^12^

A related issue pertains to the relationship between the underlying system and the target mnemic phenomena. The increase in inferential distance between the two, of course, does not entail that the hypothesized system is not causally involved in producing states of personal, autonoetic remembering. What has become increasingly evident, however, is that such production likely involves more widespread interactions with other cognitive and meta-cognitive processes, interactions that may be context-sensitive and not easily accounted for by the core explanatory principles of the new system theories. This realization motivates Mahr’s [43,47] recent attempt to account for the ways in which a general episodic simulation system produces the characteristically past-oriented, specific and self-involving memories of the personal past. Mahr reserves a key role for metacognitive monitoring mechanisms, which regularly interact with the outputs of the simulation system and whose function—particularly in attributions of ‘mnemicity’—is calibrated via an extended process of social and cultural learning [48].

The new common system theories are often presented as a natural continuation of Tulving’s work. But, we have argued, these ‘neo-Tulvingian’ theories have paradoxically come to countenance or embrace an eliminativism about episodic memory. Perhaps this should not come as a surprise. If scientific development is regulated by an ideal of theoretical unification, and is not beholden to common-sense modes of explanation, then we should expect the occasional elimination, or at least radical revision, of familiar categories [49]. Yet in this case it may be too early to admit defeat. Other recent developments in the memory sciences may indeed vindicate core ideas of *Elements*.

## 4. Back to episodic memory?

The sciences of memory constitute a broad and increasingly interdisciplinary field, housing a variety of research programmes and traditions. Empirical developments may thus motivate different responses to the common system and identity-of-process hypotheses. One critical response would involve highlighting phenomenological and behavioural differences between the target phenomena, personal remembering and imagination. Since common system theorists can allow for such differences, however, such evidence is unlikely to be decisive. What seems necessary to challenge the eliminativist verdict is evidence for dedicated mechanisms for episodic memory storage, specialized computational operations or representational kinds as well as relevant differences in underlying neural substrates. Theoretical unification under the common system and identity-of-process hypotheses can be accomplished only if there are no robust generalizations with inductive potential that pertain to a (sub)set of processes that are, in some recognizable way, distinctly mnemic in character. Even with that in mind, the sheer diversity of theoretical and empirical approaches is likely to afford different strategies against eliminativism about episodic memory. In this section, we briefly address just *two* such strategies, one negative and one positive, developing the latter more fully.

First, a key *negative* task for the critic would be to undermine confidence in the existence of a common, functionally specialized process underlying both remembering and imagination. In a recent article [50], De Brigard targets the putative role of the DMN in constructive simulation and scene construction theories of the common system. He presents evidence for the engagement of the DMN in processes that do not involve simulation of events or scenes, such as interoception [51] or semantic processing [52].^13^ Moreover, he points to the existence of spatial mappings—hypothesized to anchor event simulations—outside this network (for example [53]), to the ability of individuals with hippocampal damage to generate spatially coherent representations [54], and to the differential engagement of the hippocampus in different forms of event representations, indicating likely processing differences.^14^ This last consideration interplays well with recent neural and behavioural evidence of such differences (see below) as well as with neuroimaging data showing that, despite substantial overlap between the recollective and ‘general semantic’ networks, the hippocampus plays a hub role in the former but does not feature in the latter [16].

The *positive* task, to which we now turn, involves proposing inductively promising generalizations about mnemic processes. A key component of Tulving’s [2] account was the retention of information acquired on singular learning occasions. Recent technological advances have afforded a more direct investigation of the discrete neural vehicles underlying such retention. Molecular and transgenic ‘engram technology’ has allowed researchers to isolate neural populations coding for specific properties of learning stimuli and to demonstrate their causal relevance for subsequent retrieval, results that have reinvigorated the commitment to discrete memory traces with unique—and empirically tractable—causal histories [55,56]. Indeed, experimenters have begun to trace the (changing) neural organization of traces, showing relative diachronic stability in certain areas of the hippocampus, paired with greater cortical engagement over time [57].^15^ Engram-technological manipulations are most commonly employed in rodent conditioning studies, but there are ongoing attempts to design experimental tasks that require (more) complex representations and computational operations as well as to establish the relevance of the findings to declarative memory (for critical discussion, see [58]). In humans, the employment of representational similarity analysis and deep neural network techniques has provided insights into the structure, format and informational profiles of neural event representations [59,60]. The results show re-expression of content-sensitive patterns of neural activity at retrieval, alongside systematic transformations from perceptual to conceptual formats, raising important questions about the relation between the two processes (cf. [61]).

The studies have implicated a variety of mechanisms likely to play a role in information retention and transformation. The hippocampus, unsurprisingly, has been at the centre of attention. Different hippocampal cell types have been shown to be responsive to specific stimulus features and are believed to play important roles in binding informational content into diachronically stable event representations (for a review, see [62]). Relatedly, single neurons in the hippocampus have also been shown to respond to perceptual event boundaries, with their activity predictive of subsequent memory performance [63]. Moreover, there is some evidence for the differential role of hippocampal subfields. Cell populations in the DG and CA3 subfields have been implicated in the encoding of distinct, pattern-separated representations, affording rapid ‘episodic’ learning in the absence of interference. CA1 populations, likely involved in such learning, have also been linked to more overlapping representations, supporting information integration and generalization across events [64–66].

Behavioural evidence complements this picture nicely. Studies show systematic retention of information not only about the sensory and spatio-temporal properties of experienced events—in a characteristically ‘all-or-none’ way [67]—but also about event boundaries, with perceptual segmentation shown to affect long-term memory organization [68]. Importantly, sleep-dependent consolidation, believed to support generalization, also appears to benefit retention of information about specific events. Hence, in a memory similarity task, Hanert *et al.* [69] found that sleep in humans stabilizes pattern separation performance. Schapiro *et al.* [70] similarly reported retention of exemplar-specific information during consolidation, paralleled by improvements in memory for semantic category structure. Results of this kind suggest that discrete traces, carrying information about specific events or items, are likely often preserved in consolidation, despite documented processes of trace transformation and schematization. As Heinen *et al*. [60] point out, consolidation may involve *proliferation* of memory representations with different formats and accessibility conditions, not literal transformation of specific traces from one format to another (cf. [71]). The balance between preservation and reconstruction described in *Elements* may not be a thing of the past, after all.

Theoretical and empirical treatments of memory traces have typically paid little attention to the specific systems, and component processes, involved in information storage. This is unfortunate, as a systems perspective is of key importance: it affords the specification of the computational problems trace-manipulating systems are designed to solve as well as of their functional and resource constraints (cf. [72]). Recently, theorists have attempted to remedy this problem, with a variety of proposals about the roles discrete event representations play in cognition. There has been a characteristic emphasis on one-shot learning—i.e. ‘episodic control’—a process that involves the use of information about specific events, including actions performed, for learning an appropriate action sequence [73]. Modelling work, building on developments in deep reinforcement learning, has illustrated the computational advantages of employing episodic control processes, hypothesized to rely on dedicated hippocampal mechanisms [74,75]. In a notable recent study, Zeng *et al*. [76] provided evidence that episodic control benefits spatial learning, compared with replay-driven processes, particularly when the task is sufficiently complex and the number of learning trials is limited.

The epistemic benefits of using pattern-separated representations of individual events have also been examined in the context of associative learning, with the retention of specific details hypothesized to afford flexible use of information at retrieval [77]. Zhou *et al.* [78] examined the integration of related experiences in humans, using a hippocampally dependent associative inference task. Their results indicated that subjects likely employ both pattern-separated and integrated event representations, with characteristic advantages in different retrieval contexts. Specifically, the retention of pattern-separated representations was found to be beneficial in circumstances that do not require rapid recognition of item relatedness, for example in explicit inference. (In contrast, integration of experiences via interleaved learning gave rise to fast, automatic recognition.) Comparing computational models of memory integration, Zhou *et al*. concluded that only models that include both kinds of representations can account for the available behavioural data.

The evidence presented above motivates a careful reassessment of the identity-of-process view. A tentative hypothesis posits the existence of a set of episodic *memory* processes—possibly constituting a memory system—dedicated to the preservation, maintenance and retrieval of discrete event representations. Tightly integrated, the processes are relatively limited in their ability to generate novel information, yet afford flexible and context-sensitive recall. The preservation of discrete memory traces is constitutively involved in remembering the personal past, with information from first-hand experiences systematically prioritized at retrieval. Such preservation, to echo Tulving & Watkins, ‘is a necessary condition for the subsequent retrieval of information about the event’ [79, p. 261]. Despite the substantial similarities—plausibly due to the engagement of simulative processes of event representation—discrete memory traces are likely *not* constitutively involved in future-oriented or counterfactual imagination. While these often rely on individual traces, their production is not ‘diachronically’ constrained in the same way ([44]; cf. [80]). Additional support for this hypothesis may come from more recent studies comparing the neural underpinnings and development of remembering and episodic future thought. Building on prior work, Østby *et al*. [81] found that the functional connectivity of the DMN, in adolescents and young adults, was strongly related to the subjective quality of personal remembering, yet only marginally related to episodic future thought. This finding fits well with the growing number of studies reporting significant differences in the developmental trajectories of the two capacities (for a review, see [82]).

Importantly, positing a specialized set of processes for the preservation of event representations does not commit us to an ‘archival’ or non-constructivist view of episodic memory. As we have tried to emphasize throughout the paper, memories emerge via the systematic interaction of a variety of processes. While preservative processes may be limited in their capacity to generate novel information, such generation occurs regularly via synergistic trace–cue interactions. Moreover, event representations with different formats and informational profiles are likely systematically maintained and manipulated, from encoding to consolidation and retrieval. These representations may be stored independently, yet they contribute jointly to the informational structure of individual memories, with their dynamic interactions dependent on a number of factors, such as attention, time, task demands and prior knowledge [4,60,71,83].

While there are reasons for optimism about the place of episodic memory in science, we should proceed with caution. Process hypotheses owe answers to notoriously difficult questions about the individuation of processes and their integration in distinct neurocognitive systems. Individuating a computational process requires careful, and likely theory-laden, abstraction from the ‘unfiltered’ causal structure of the world [84], an endeavor that has proven difficult across the memory sciences [85]. Questions about integration, which had worried Tulving throughout his career, are arguably even more pressing. While functionally specialized, memory systems were hypothesized to have ‘fuzzy boundaries, have overlapping constituent processes, and [to] interact with one another in intricate ways’ [7, p. 18]. Establishing whether a set of processes are sufficiently integrated to constitute a system requires specification of the degree of integration necessary for constituency, with pragmatic factors likely to play a major role. Further, the integration of a set of processes in a common neurocognitive system might essentially depend on the experiences of the organism, thus constituting a kind of developmental achievement [86]. Getting reliable evidence for the actual form and degree of integration is difficult, particularly given the hypothesized intricate interactions between systems. While emerging neuroimaging and network data may alleviate some of these problems, establishing the proper interpretation and relevance of such data remains a considerable challenge (for a discussion of these issues, see [9]).^16^

A proper assessment of the rival views, and of their consilience with the ideas outlined in *Elements,* thus requires navigating complex empirical and conceptual terrains. Future work, aiming to advance the debate, would have to examine the architectural connections between ‘core’ episodic processes (such as information retention, constructive simulation and autonoesis), their possible integration in neurocognitive systems, and the principles that govern their interactions in the production of mnemic and imaginative states. Such work, the methodological underpinnings of which may already be in place [16,17,43], promises to be arduous and will plausibly involve a further increase in inferential distance between the relevant explanantia and explananda. Yet we have no other choice. Engaging in this work is the only way to hasten the arrival of a future in which we know whether Tulving’s [2] signature idea of a specialized episodic memory system is wrong—and perhaps even whether it is right.

## 5. Conclusion

The notion of episodic memory has proven remarkably resilient. Somewhat surprisingly, however, this resilience may not be a reliable indicator of its good fit with nature. For Tulving [2], the notion stood for a hypothesized neurocognitive system underlying the familiar experience of remembering the personal past. In the intervening decades, empirical developments have necessitated significant amendments to this picture. Keeping the systems perspective, contemporary theories posit a common system for remembering and imagining, often retaining the notion of episodic memory to refer to individual memories produced by this system. In this paper, we examined this shift in perspective, diagnosing the new theories as eliminativist in tendency. On many common system views, we argued, episodic memory does not constitute a real kind, appeals to which may ground hypotheses with inductive potential. We then moved on to examine and systematize recent evidence that may challenge this conclusion. Such evidence comes from a number of research programmes and provides preliminary support for the existence of a set of processes, dedicated to the retention and maintenance of discrete representations of personally experienced events. Future work, which will have to negotiate difficult conceptual and empirical questions, will reveal how these processes are involved in remembering and imagination and whether they constitute a unified episodic memory system.

## Data Availability

This article has no additional data.

## Footnotes

^1^
The notion of episodic memory first appeared in Tulving [6] in the context of the hypothesis that episodic and semantic memory are distinct information-processing systems with characteristic computational properties. At that point, the hypothesis was treated as a ‘pretheoretical position whose major usefulness may turn out to lie in facilitating theory construction’ [6, p. 384] and was not explicitly linked to a search for systems or natural psychological kinds.

^2^
For the purposes of this paper, we do not want to commit to any particular conception of natural kinds. While we have sympathies for Boyd’s [10] homeostatic property cluster view, we have some reservations about the necessity of a ‘homeostatic’ mechanism that keeps the relevant properties clustered. A loosening of Boyd’s account, perhaps along the lines of Khalidi [11], seems to us a step in the right direction.

^3^
The immediacy of awareness entails the absence of explicit inference, *not* immunity to error. Subjects undergoing autonoetic experiences can, of course, be mistaken about (having previously experienced) a past event.

^4^
Technically, it is *ecphory*—a process of activation of a latent trace via conversion of trace and cue information—that is synergistic. Ecphory is only one element of retrieval [2, pp. 175–178]. So as not to complicate things too much, we gloss over this issue in the main text.

^5^
Space prohibits a detailed investigation of the claims advanced in this section. For a fuller historical and philosophical treatment of the original theory in relation to the empirical research that it motivated and rested on, see [9].

^6^
K.C.’s general world knowledge, including much of his knowledge about himself, was surprisingly preserved, which suggested that the amnesia was characteristically *episodic*. For more details, see Rosenbaum *et al*. [19].

^7^
Remembering and imagining activated the DMN relative to a semantic control task, such as thinking about the definitions of cue words and generating words related to the cue. The DMN is thought to be involved in a number of different tasks including when individuals are not engaged in a cognitive activity that requires attention to external sources, or when individuals are mind-wandering [26]. We discuss the DMN further below.

^8^
Addis [33, p. 234] notes that the DMN is not only involved in forms of mental time travel, but is also engaged in other forms of cognition including creativity, theory of mind, narrative comprehension and event perception. These diverse forms of cognitive activity are nonetheless united, she thinks, by involving simulations—the mental renderings of experience.

^9^
See also, for example De Brigard [39, p. 173]; Addis [33, p. 239]; Schacter [42, p. 40]; Mahr [43, p. 3].

^10^
We assume, of course, that none of these states are underlaid by dedicated brain systems.

^11^
Michaelian [46] offers a comparably radical revision, arguing that episodic remembering is constituted by simulation of a past event, regardless of whether the event has been previously experienced.

^12^
Our reviewers suggest that theorists, faced with these difficulties, may shift to a pragmatist or normative conception of episodic memory. We agree. Indeed, both descriptive and normative perspectives may be appropriate in different explanatory contexts [3,5]. Here, however, we examine the development of ‘neo-Tulvingian’ theories which purport to inherit the methodological commitments of Tulving’s multiple memory systems approach and treat episodic memory as a *natural* kind.

^13^
De Brigard raises further worries about understanding the DMN as the simulation system of the brain. He notes that many non-human animals possess a DMN but thinks it is highly unlikely they engage in complex episodic simulations. He also emphasizes that not all episodic constructive processes depend to the same extent on core regions of the DMN; for example, the DMN fails to be preferentially recruited in cognizing episodic counterfactual situations that involve objects as opposed to people, and it is also not recruited when thinking counterfactually about what an unfamiliar person in an unfamiliar situation would have done.

^14^
Importantly, subjects with hippocampal damage exhibit substantial deficits in episodic *memory* tasks, despite their preserved ability to generate spatially coherent representations (see the discussion in [54]). These deficits are plausibly (partially) due to impairments of specialized preservative processes of the kind we examine below.

^15^
Specifically, Refaeli *et al*. found that a core ensemble in the CA1 region of the dorsal hippocampus was *necessary* for retrieval of both recent and remote memories.

^16^
On Tulving’s conception, memory systems are constituted by tightly integrated constituent computational processes. Hence, positing a memory system requires evidence not only of specialized processes (which may or may not be proprietary to the system) but also of their systematic integration. As we have tried to emphasize, the latter kind of evidence may be particularly difficult to obtain. We thank a reviewer for prompting us to add this clarification.
